# Polycystic Ovarian Syndrome (PCOS): Does the Challenge End at Conception?

**DOI:** 10.3390/ijerph192214914

**Published:** 2022-11-12

**Authors:** Fadi G. Mirza, Muna A. Tahlak, Rachelle Bou Rjeili, Komal Hazari, Farah Ennab, Charlie Hodgman, Amar Hassan Khamis, William Atiomo

**Affiliations:** 1 Latifa Women and Children Hospital, Dubai P.O. Box 9115, United Arab Emirates; 2 Department of Obstetrics and Gynaecology, College of Medicine, Mohammed Bin Rashid University of Medicine and Health Sciences, Building 14, Dubai Healthcare City, Dubai P.O. Box 505055, United Arab Emirates; 3 Department of Obstetrics and Gynaecology, College of Physicians and Surgeons, Columbia University, New York, NY 10032, USA; 4 Faculty of Medicine, American University of Beirut, Beruit P.O. Box 11-0236, Lebanon; 5 School of Biosciences, University of Nottingham, Loughborough LE12 5RD, UK

**Keywords:** polycystic ovary syndrome, PCOS, pregnancy, obstetric, complications, obesity, miscarriage, diabetes, preterm, hypertension

## Abstract

Polycystic ovary syndrome (PCOS) is a prevalent condition that not only has the potential to impede conception but also represents the most common endocrine dysfunction in fertile women. It is considered a heterogeneous and multifaceted disorder, with multiple reproductive and metabolic phenotypes which differently affect the early- and long-term syndrome’s risks. Undoubtedly, the impact of PCOS on infertility has attracted most of the attention of healthcare providers and investigators. However, there is growing evidence that even after conception is achieved, PCOS predisposes the parturient to several adverse pregnancy outcomes including a high risk of pregnancy-induced hypertension, spontaneous abortion, gestational diabetes, preeclampsia, and preterm birth, which increase the risks of stillbirth and neonatal death. Fetal growth abnormalities may also be more common, but the relationship is less well defined. This narrative review aims to summarize current knowledge regarding these conditions as they interplay with PCOS and concludes that although there appears to be an increase in these complications during the pregnancy of women with PCOS, there is a need for further research to clarify the possible confounding impact of obesity. Implications for clinical practice and future research are outlined.

## 1. Introduction

Polycystic ovary syndrome (PCOS) is the most common diagnosed endocrine disorder in women [1] and a major cause of anovulatory infertility. The prevalence is thought to range from 4% to 26% depending on the population studied [2,3,4]. PCOS patients can present a wide range of signs and symptoms, making it difficult to agree on the precise definition of the condition. The diagnosis of PCOS is currently based on the criteria of the ESRHE/ASRM Rotterdam consensus meeting in 2003 [5], which broadened the previous NIH (National Institute of Health) classification of 1990. It is based on at least two of the following features: oligo-anovulation, hyperandrogenism, and polycystic ovaries by ultrasound. In 2006, the Androgen Excess Society (AES) set up a committee of experts to review all the data published on PCOS for the purpose of simplifying diagnoses [6]. The AES criteria require clinical and/or biochemical hyperandrogenism simultaneously with oligo/anovulation and ultrasonographic evidence of polycystic ovaries.

Although the etiology of PCOS is not completely understood yet, PCOS is considered a multifactorial disorder with various genetic, metabolic, endocrine, and environmental abnormalities [7]. There is increasing evidence suggesting that PCOS affects the whole life course of a woman and can begin in utero in genetically predisposed subjects; manifest clinically at puberty with menstrual disorders; and continue during the reproductive years with ongoing menstrual disorders, infertility, and obesity. Early diagnosis is therefore crucial by enabling close follow-up to reduce the risks of potential long-term complications such as type 2 diabetes, hypertension, and endometrial cancer [8]. It is now widely recognized that insulin resistance, manifesting above all in obese or overweight women, but often also in lean PCOS women, is one of the key factors in this complex disorder. Insulin resistance is defined as the requirement of excessive insulin for its metabolic activities [9,10,11]. It is thought to underpin hyperandrogenism by acting synergistically with luteinizing hormone (LH) on ovarian steroidogenic enzymes and on sex hormone-binding globulin (SHBG) production by the liver [12]. However, there is growing evidence that even after conception is achieved, PCOS predisposes the parturient to several adverse pregnancy outcomes. The latter include spontaneous abortion, gestational diabetes, hypertensive disorders of pregnancy, preterm birth, and possibly fetal growth abnormalities (Table 1).

Although it is important to treat the short-term disturbances in women, research shows that it is important to think about the maternal and fetal outcomes of women with PCOS during a pregnancy [30,31]. Here, we present an overview of these conditions as they interplay with PCOS and argue that although there appears to be an increase in some complications during the pregnancy of women with PCOS, there is an urgent need for further research to clarify the possible confounding impact of obesity on these reported complications in PCOS. Implications for clinical practice and future research are also outlined.

The papers included in this narrative review were identified primarily through PubMed, and relevant secondary references were obtained following reading the primary publications. The following keywords were used “PCOS” AND “Pregnancy”, “PCOS” AND “miscarriage”, “PCOS” AND “gestational diabetes”, “PCOS” AND “hypertension in pregnancy”, “PCOS” AND “Pre-eclampsia”, “PCOS” AND “Preterm Birth”, “PCOS” AND “Fetal growth”. Searches retrieved were sorted by date of publication and the abstracts initially read. Full texts were then retrieved if the title and abstracts indicated that the paper referred to pregnancy complications. References of relevance were then stored on the Mendeley reference manager. The paper followed the Scale of Assessment of Narrative Review Articles (SANRA) [32].

## 2. Spontaneous Miscarriage

A miscarriage is defined as the loss of a pregnancy before 24 weeks gestation or the loss of a fetus weighing less than 500 grams. It is thought to affect 15% of clinically recognized pregnancies with devastating psychological consequences. Spontaneous miscarriages are often associated with chromosomal abnormalities in the fetus; however, miscarriages can also be associated with maternal and fetal infection, maternal obesity, advanced maternal age, structural abnormalities in the fetus and uterus, systemic illnesses in the mother and endocrine, metabolic, and clotting disorders in the pregnant mother.

A link between PCOS and spontaneous miscarriage has been thought to be present for over three decades; however, determining the precise degree of risk and underlying mechanisms to inform prevention and treatment remains a challenge. Early associations are found in reports by Roy Homborg and colleagues in 1988, stating that women with PCOS who were resistant to clomiphene, often suffered from early pregnancy loss. This was thought to be caused by the raised basal luteinizing hormone (LH) levels in women with PCOS in a study of 54 infertile women with PCOS [14] where basal LH levels were significantly lower in pregnancies which progressed compared with pregnancies which were complicated by miscarriage [7]. The association between PCOS and miscarriage was also thought to include a predisposition to recurrent miscarriage, following a study by Sagle et al. in 1988 [33], who found that 82% of regularly menstruating women with recurrent miscarriage had ultrasound evidence of polycystic ovaries, and another study by Clifford et al. in 1994 [34], who found polycystic ovaries were present in 56% of 500 women with recurrent miscarriage.

This link between PCOS, miscarriage, and LH hypersecretion, however, began to be thrown into doubt when a subsequent study in which raised LH levels were suppressed before pregnancy in a randomized controlled trial of 106 women with a history of three or more consecutive first trimester miscarriages, polycystic ovaries, and hypersecretion of LH did not find an improvement in pregnancy outcome [35]. Other potential mechanisms for the link between PCOS and miscarriage have therefore been investigated, including the role of insulin resistance [36], hyperhomocysteinaemia, hyperandrogenemia, the plasminogen activator inhibitor-1 [16] vitamin D-binding protein [17] and obesity. For example, a study conducted in India [15] aimed to assess the role of hyperhomocysteinemia and insulin resistance in PCOS patients, and how they may be related to recurrent pregnancy loss. The investigators enrolled two groups of patients, a PCOS and a non-PCOS group, each of which was further subdivided into 4 categories: hyperhomocysteinemia, normohomocysteinemia, insulin resistance, and non-insulin resistant. The rate of spontaneous abortion in both the PCOS-hyperhomocysteinemia and the non PCOS-hyperhomocysteinemia subgroups was significantly higher than the normohomocysteinemia groups, irrespective of PCOS or not. The authors concluded that hyperhomocysteinemia played a considerable role in early pregnancy loss and suggested that the higher risk of spontaneous abortion observed in women with PCOS was likely to be due to their high prevalence of obesity (which is associated with hyperhomocysteinaemia) and the type of treatment they received.

The association between PCOS and miscarriage has, however, not always been present in research studies. For example, a meta-analysis of studies concerning women with and without PCOS undergoing IVF demonstrated no difference in miscarriage rates [37]. Additionally, none of the nine studies included in another meta-analysis [38] showed any difference in miscarriage rates between PCOS and non-PCOS women. A PCOS consensus statement in 2012 also suggested that miscarriage rates were comparable to women without PCOS; however, the available data showed conflicting results [39]. The uncertainty in the association between PCOS and miscarriage is further illustrated in a large Australian study that found that although the miscarriage rate was significantly more frequent in women with PCOS than in controls (20 versus 15%), PCOS was not an independent risk factor for pregnancy loss, but the miscarriage rate was strongly influenced by BMI [13]. More recent studies also continue to fuel this uncertainty. For example, a recent study from India [40] found that spontaneous abortion rates were higher in 51 women with PCOS compared with controls. However, PCOS women were older and had a higher body mass index (BMI) which could potentially have been confounding variables, just as increased age and obesity are associated with miscarriage. Another study by Sha et al. [41], in a meta-analysis of pregnancy-related outcomes and complications in women with polycystic ovary syndrome undergoing IVF found that women with PCOS had higher risks of miscarriage (OR 1.41); however, the authors concluded by stating that they were unable to perform a further analysis to evaluate the influence of phenotypic variants of PCOS on the prevalence of pregnancy and neonatal complications. Finally, an expert opinion piece published in 2022 on the association between PCOS and miscarriage stated that women with PCOS are at increased risk for ectopic pregnancy, hydatidiform molar pregnancy, and miscarriage, mainly following fertility treatments, but that given that fertility treatments are known risk factors for the outcomes, it is hard to disentangle the association of PCOS per se with these adverse outcomes [42].

The link between PCOS and miscarriage therefore remains unclear and further prospective observational studies are required.

## 3. Gestational Diabetes Mellitus

Gestational diabetes mellitus (GDM) is a form of diabetes first recognized in the second trimester of pregnancy. In Europe, the prevalence of GDM is around five percent [19]. However, the prevalence is steadily increasing, as maternal obesity is a major predisposing factor for GDM, and more women of child-bearing age are overweight or obese [20]. The risks of GDM include an increased risk of large babies, caesarean sections, birth trauma, still births, and neonatal jaundice. It is also thought to increase the risk of future type 2 diabetes. Apart from hyperglycemia, GDM is associated with increased oxidative stress and inflammation in the placenta and fetus [20,21]. GDM also increases trans-placental fatty acid and lipoprotein transport and turnover [22,23]. Previous research also shows that GDM causes major changes in the composition of the fetal lipoprotein proteome [43], and that fetal lipoprotein functionality is different from adults [44]. It is therefore important to identify women at risk of GDM so that appropriate management can be arranged in pregnancy.

The possible confounding impact of obesity on the risks of PCOS and GDM are however uncertain. One of the early publications on a possible link between PCOS and GDM was by Gjonnaes, from Norway, in 1989 [21]. In a study of 89 women with PCOS who conceived after ovarian electrocautery, the pregnancy continued beyond 31 weeks in 62. In this group, the frequency of diabetes was 8.1%. Although no control frequency was provided, it is interesting to note that an increased frequency of GDM was reported as confined to overweight women, which begs the question as to what extent any associations between GDM and PCOS are independent of obesity. These findings were similar to a study published in 1990 [45], in which the incidence of glucose intolerance in 76 patients with PCOS who became pregnant after treatment for ovulation was compared with that in 95 healthy women, matched with respect to age and diabetic background, who conceived without intervention. The study found that the incidence of abnormal glucose tolerance test results in the treated group was twice that in the normal group (19.7% vs. 9.5%). However, a study, published the following year, in 1991 [46], found that the incidence of gestational diabetes in PCOS patients was similar to the frequency in the controls, and the authors concluded that preexisting PCOS did not appear to increase the risk of developing gestational diabetes.

Ongoing uncertainty about the risks of GDM in PCOS and possible confounding effects of obesity were also present in later studies. For example, in a study from Finland in 2001 [18], 99 pregnancies were retrospectively evaluated in women with PCOS, and the findings were compared with an unselected control population. The average body mass index (BMI) in PCOS patients was greater than that in controls, and GDM developed in 20% of the PCOS patients and in 8.9% of the controls. However, BMI >25 seemed to be the greatest predictor for GDM. Similar findings were present in a study published in 2003, in which the pregnancy records of 38 PCOS patients were compared retrospectively with 136 non-PCOS patients randomly in a study by Turhan et al. [47], from Turkey. The prevalence of gestational diabetes mellitus (GDM) was similar in both groups, raising questions as to the risk of GDM in PCOS.

The association between PCOS and GDM was, however, then examined in subsequent meta-analyses. Boomsma et al. [48] for example, in 2006 undertook a meta-analysis of pregnancy outcomes in women with PCOS involving 720 women presenting with PCOS and 4505 controls. Women with PCOS demonstrated a significantly higher risk of developing gestational diabetes with an odds ratio of 2.94. There was, however, no investigation of the possible confounding impact of obesity. Toulis et al. [49], in a subsequent meta-analysis in 2009, found that women with PCOS demonstrated a significantly higher risk for the development of GDM as compared to women without PCOS with an odds ratio of 2.89. The authors, however, found significant statistical heterogeneity and concluded that the significant heterogeneity among studies and dependence of the outcome on the study type made the higher risk of GDM in women with PCOS a questionable finding, thus calling for the conduction of properly designed studies before any recommendation to pregnant women with PCOS regarding the risk of GDM. 

In addition to these meta-analyses, individual studies continued to investigate the link between PCOS and GDM, with some concluding that PCOS was an independent risk factor for type 2 diabetes in fertile women with GDM [50], and others indicating that obesity was a confounding variable. For example, in 2011, a study in South Korea investigated 1339 patients, all presenting for assisted reproductive technology for infertility either due to PCOS or tubal factors [29]. The patients were divided into four groups, obese and non-obese PCOS patients, and obese and non-obese patients with infertility secondary to tubal factors. Interestingly, GDM developed in fewer (1.1%) non-obese women with PCOS compared to 1.8% of non-obese women in the control group, and GDM was higher in the obese groups, regardless of whether they had PCOS or not. 

On the other hand, two studies from China published in 2011 [51] and 2019 [52] specifically examined the risk of GDM in PCOS and both found that normal weight/BMI women PCOS also had an increased risk of GDM, although subsequent studies infer a possible confounding effect of obesity [53,54]. A meta-analysis published in 2022, however, concluded that although PCOS was significantly associated with an increased risk of GDM, the role of several potential confounding variables including BMI, the severity of GDM, and serum lipid level could not be evaluated [55].

This controversy about the role of possible confounding factors in the risk between GDM and PCOS unfortunately therefore remains, with a recent expert opinion [42] concluding that the higher risk of GDM was independent of, yet exacerbated by, obesity in PCOS related to insulin resistance, in contrast to three relatively recent publications including two meta-analyses and a Danish prospective study which all [56,57,58] concluded that PCOS may not be an individual risk factor for GDM as pregnancies in PCOS are characterized by factors known to increase the risk of GDM, especially high BMI and fertility treatment.

### Mechanisms

The key mechanism thought to underpin the possible increased GDM risk is insulin resistance. As pregnancy occurs, various placental hormones get released and this eventually leads to a physiological state of insulin insensitivity [19]. The different placental hormones increase the absorption of nutrients to promote fetal growth, but when this process occurs in women with PCOS who are already predisposed with a background of insulin resistance, the development of GDM becomes almost inevitable. Additionally, the overexpression of inflammatory mediators, alongside a remarkable rise in reactive oxygen species (ROS), could potentially cause further metabolic changes leading to the inhibition of the insulin secretion pathway, and consequently lead to reduced beta-cell insulin secretion and GDM. 

However, as insulin resistance is related to obesity, it has not always been possible in all studies to identify an increased risk for GDM in PCOS. Maternal insulin resistance is a physiological process which develops during gestation to ensure adequate fetal energy supply. Whereas most women can cope with this metabolic adaptation, some develop GDM. Another potential mechanism involved in the association between PCOS and GDM is low vitamin D levels [20]. Women with PCOS have been found to be deficient in vitamin D, particularly those with a higher weight. Hypovitaminosis is a risk factor for glucose intolerance, and reduced levels of vitamin D are associated with insulin resistance and increased diabetes risk. The interlink between obesity and vitamin D [59], however, makes it challenging to exclude the possible confounding impact of obesity on GDM risk in PCOS.

In summary, these studies on the link between PCOS and GDM reveal that although PCOS is thought to be linked with GDM, the possible confounding effects of obesity on the increased risk of GDM in PCOS need to be explored further.

## 4. Hypertension in Pregnancy (Preeclampsia)

Hypertensive disorders of pregnancy are significant causes of maternal and fetal death, and they are the third highest cause of maternal death globally. It is therefore crucial that women at risk are identified early and carefully managed during pregnancy. The American College of Obstetricians and Gynecologists Task Force on Hypertension in Pregnancy defined chronic hypertension as a systolic blood pressure of 140 mmHg or more or a diastolic blood pressure of 90 mmHg or more on two separate occasions at least 2 hours apart occurring before pregnancy or developing before 20 weeks of gestation during pregnancy. Obesity is also a risk factor for hypertension in pregnancy, and as with GDM, there is some debate about the confounding role of obesity in the link between PCOS and hypertension in pregnancy.

Historically, as with GDM, the study [21] by Gjonnaes, from Norway, in 1989 was one of the early studies to link hypertensive disorders in pregnancy with PCOS. In the study, the frequency of preeclampsia was 12.9. However, as with GDM, the increased frequency was confined to overweight women. Subsequent publications in 1998 [60] and 1999 [61] also supported an association between PCOS and hypertensive disorders of pregnancy. In the study published in 1998, de Vries and colleagues performed a case control study involving a retrospective analysis of 81 patients with PCOS, consecutively becoming pregnant during a seven-year period. Each PCOS patient was matched for age and parity with one control patient. The incidence of preeclampsia was significantly higher in patients with PCOS than in controls, and it was thought that the higher incidence could not be explained by body mass index, endocrine profile before pregnancy, induction of ovulation, or treatment regimens. Similarly, Fridström and colleagues in 1999, in a retrospective case-control study, compared 33 women with PCOS and 66 women without PCOS. They found that there were no differences in blood pressure during the first and second trimester. However, during the third trimester and labor, the PCOS group had a significantly higher blood pressure than the control group. 

Following these early studies, other studies summarized in large meta-analyses, each reviewing more than 20 studies and review articles, also revealed a significant increase in both pregnancy-induced hypertension (PIH) and preeclampsia in PCOS patients as compared to controls. In a meta-analysis published in 2006 [48] including 720 women presenting with PCOS and 4505 controls, women with PCOS demonstrated a significantly higher risk of developing pregnancy-induced hypertension (OR 3.67) and preeclampsia (OR 3.47). Similar findings were also present in subsequent meta-analyses published in 2011 [62] and 2013 [63], with one [63] of them involving 4982 women with PCOS and 119692 controls showing that women with PCOS had a 3.43 and 2.17 times increased risk of hypertension in pregnancy and preeclampsia, respectively, compared to controls. These findings were also reflected in a literature review by Palomba et al. [38] in which the data from three meta-analysis found that PCOS patients had a 3-4-fold increased risk of developing PIH or preeclampsia. The review, however, concluded with an acknowledgment that available studies suggested that PCOS-related features including obesity may increase the risk of pregnancy complications in PCOS.

As with GDM, obesity may therefore confound the association between PCOS and hypertension in pregnancy as consistent with the following studies. A study by Han et al. [29], which compared obese PCOS patients, non-obese PCOS patients, obese controls, and non-obese controls noted no difference in the prevalence of PIH in any of the four groups. A further study by Naver et al. [64], that compared singleton pregnancies in PCOS patients from a private fertility clinic to a background population of singleton pregnancies from a local hospital adjusted for age, parity, and BMI concluded that the risk of preeclampsia in the PCOS group was no different from the chosen background population. However, when comparing the hyperandrogenic subgroup to the background population, they had a significantly increased risk of preeclampsia during pregnancy, implying that hyperandrogenemia was possibly a better predictor for hypertension in pregnant women with PCOS. On the other hand, a meta-analysis published in 2021 [65] including 30 studies found that PCOS was associated with a higher risk of hypertensive disorders of pregnancy (OR 2.02) including pregnancy-induced hypertension (OR 1.94) and preeclampsia PE (OR 2.07), with the association remaining high after adjusting for body mass index (BMI) and the increased risk of hypertensive disorders of pregnancy for the PCOS group remaining significant in subgroups of BMI. 

### Mechanisms

A range of mechanisms has been proposed as potentially underpinning the association between PCOS and hypertension in pregnancy. Some of these include insulin resistance and hyperhomocysteinaemia. A more recent development is in the possible role of co-enzyme A restriction [24] in the link between PCOS and hypertension in pregnancy. However, the potential confounding role of obesity is unclear. Many studies have suggested that hyperinsulinemia, which is present in PCOS, is the main pathogenic mechanism of hypertension in pregnancy, as the vascular endothelial cells which are insulin-sensitive are activated due to this state of hyperinsulinemia, causing a decreased production of prostaglandin, and thereby increasing the peripheral vascular resistance leading to an increase in the blood pressure [66]. It was also noted that vascular lumen stenosis and endothelial dysfunction are also responsible for PIH in patients with PCOS. This is largely because these women have increased levels of hyperlipidemia [67].

For example, in a study published in 2002 [22], pregnancies and neonatal outcomes were recorded in a prospective cohort study comprising 29 non-insulin-resistant PCOS women, 23 insulin-resistant PCOS women, and a control group of 355 women who had conceived after assisted reproduction. Hypertension, preeclampsia, and GDM were recorded as well as pregnancy duration, method of delivery, and birth weight. The frequency of hypertension was significantly elevated in PCOS women (11.5%) compared to controls (0.3%). However, the frequency of preeclampsia was significantly elevated only in the insulin-resistant PCOS women (13.5%) compared to controls (7.0%). Insulin resistance in PCOS is also associated with hyperhomocysteinaemia, which has been found to be associated with pregnancy-induced hypertension [23]. Furthermore, a study [68] of 155 infertile patients with PCOS and 100 women serving as controls found that mean plasma homocysteine in the PCOS group was significantly higher than in the control group and significant correlations were found between all insulin resistance indices and homocysteine levels. A multiple logistic regression analysis found insulin resistance was the major factor that influenced homocysteine levels. In a recent study by Hodgman et al. in 2021 [24], investigating preeclampsia data from molecular up to population scales, the results pointed to a shortage of co-enzyme A (CoA) as a feature leading to the production of signaling lipids and toxic metabolites that induced vasoconstriction, inflammation, oxidative stress, thrombogenesis, acidosis, apoptosis, and neurotoxicity. It proposed that the elevated levels of unsaturated triglycerides present in the plasma of PCOS patients depress CoA levels further by thioesterification, promote oxidative stress by stimulating peroxisome formation, and may lead to obesity. Clinical studies to validate these findings and investigate the possible confounding impact of obesity are however required.

In summary, although PCOS is thought to be associated with pregnancy-induced hypertension and preeclampsia, more original clinical studies are required to test this hypothesis, investigate the underlying mechanisms, and particularly decipher the interplay between obesity and hypertension in pregnant women with PCOS.

## 5. Preterm Birth

Preterm birth (PTB), birth at <37 weeks of gestation, is a significant global public health problem. Worldwide, about 15 million babies are born preterm each year resulting in more than a million deaths of children. Preterm infants are at increased risk of developing neonatal and long-term complications. Thus, identifying women at risk of PTB at an early life stage may allow early health monitoring and intervention. One of the early studies on the association between PCOS and preterm births was by Mikola et al. in 2001 [18]. In the study, 99 pregnancies were retrospectively evaluated in women with PCOS, and the findings compared with an unselected population. Premature delivery occurred in 16.1% of PCOS patients compared with 6.5% of the control population; however, this was thought to be largely explained by multiple pregnancies. The BMI was, however, greater (25.6 versus 23) in the PCOS women compared with controls, which again begs the question as to whether BMI explains the increased premature delivery rates as with the previous pregnancy complications discussed in the preceding sections of this manuscript.

Subsequent retrospective and cohort studies shortly after were, however, conflicting, with some showing an association between PCOS and preterm births and others not. For example, a retrospective study in 2003 [69] evaluated the pregnancies of 66 women with PCOS who had been treated for infertility compared with a group of 66 age- and weight-matched controls and did not find any significant differences in the prevalence of premature deliveries between the group of PCOS patients and the controls. Similarly, a retrospective study on Asian women with PCOS published in 2006 [25] concluded that PCOS was not a demonstrable risk factor for premature birth. In the study, 47 pregnancies in 41 PCOS women were compared with 264 pregnancies in 222 women with normal menstruation. Although the prevalence of premature delivery was significantly greater in PCOS women (13.3%) than in the controls (5.4%), the mean body mass index (BMI) and proportion of BMI of >25 kg/m^2^ were significantly higher in the PCOS than in the control group, suggesting that this might have been an important confounding variable.

The uncertainty in the literature about the association between PCOS and preterm birth led to the publication of a systematic review in 2006 by Boomsma et al. [48], in which 15 of 525 identified studies were included, involving 720 women presenting with PCOS and 4505 controls. The results showed that women with PCOS were found to have a significantly higher risk of developing preterm birth (OR 1.75). However, this difference was small, and when the mean length of gestation was analyzed, no significant difference was observed. To minimize the effect of confounding factors frequently associated with PCOS, such as BMI, the authors identified studies in which these factors did not differ between the PCOS and control populations.

However, over the years, other studies since 2006 have continued to investigate the link between PCOS and preterm births, concluding that PCOS was associated with preterm delivery [70,71,72]. For example, a large historic cohort study of pregnant women including those who conceived spontaneously and through assisted reproductive technology was performed at Guangzhou Women and Children’s Medical Center (GWCMC), China, between 1 January 2011, and 31 December 2014, to assess the risk for adverse birth outcomes among Chinese women with PCOS [73]. This study showed that women with PCOS had an increased risk of preterm birth. Similarly, a large population-based cohort study found that PCOS was strongly associated with preterm birth [74], and when the authors adjusted for BMI, and the association between PCOS and preterm birth, the association was only slightly attenuated, suggesting that the difference in BMI between PCOS and non-PCOS women did not completely explain the association between PCOS and preterm birth. A further systematic review published in the Lancet also showed that PCOS increased the risk of preterm birth by at least 2-fold [75].

A recent population-based study conducted in the UK [26] also found that maternal PCOS was associated with an increased risk of preterm and caesarean delivery. The authors were, however, unable to evaluate any effect modification attributable to in vitro fertilization when assessing the association between PCOS and the risk of obstetric outcomes or adjusting for pregnancy-induced complications or gestational weight gain. The study authors therefore concluded that it was possible that pregnancy complications (for example, hypertension, multiple pregnancy, and caesarean sections) formed the interlink between maternal PCOS and the risk of preterm delivery, given the increased risk of preterm delivery conferred by these pregnancy complications. The latest systematic review on the association between PCOS and preterm birth aimed to clarify the relationship between pre-pregnancy overweight/obesity with PCOS and subsequent pregnancy outcomes including preterm birth. A total of 16 cohort studies, including 14 retrospective cohort studies (n = 10,496) and another 2 prospective cohort studies (n = 818), contributed to a total of 11,314 women for analysis. The authors did not find any association between pre-pregnancy overweight/obesity women with PCOS and preterm birth, suggesting that factors other than obesity on its own may modulate any links between PCOS and preterm birth, if these are truly present [76].

### Mechanisms

Various mechanisms have been suggested as potential mediators of the risk of preterm delivery and PCOS including obesity, the increased incidence of multiple pregnancies and nulliparity, the associated increased estrone levels, hyperinsulinemia, and the subsequent diabetic and hypertensive predispositions. In previous reports, women with PCOS often required assisted reproductive technology to become pregnant, increasing the risk of multiple births and hypertensive disease, which are associated with preterm birth. The association between PCOS and preterm birth may thus be an interaction with assisted reproductive technology. Interestingly, Feigenbaum et al. [27] examined the prevalence and incidence of cervical insufficiency in PCOS pregnant women. After controlling for maternal age, nulliparity, ethnicity, BMI, and fertility treatment, both the prevalence and incidence of cervical insufficiency were significantly elevated in PCOS pregnant patients when compared to non-PCOS pregnant women. Other mechanisms have also been proposed including increased levels of pro-inflammatory proteins, tumor necrosis factor (TNF), and hyperandrogenism.

In summary, although the risk of preterm delivery in PCOS appears to be increased, the mechanisms, including the role of potential confounders such as other pregnancy complications, obesity, inflammation, cervical incompetence, hyperandrogenism, and obesity require further research.

## 6. Fetal Growth Abnormalities

Fetal growth is an important indication of fetal health as abnormalities may indicate a range of adverse maternal conditions, and it can be associated with increased neonatal morbidity and mortality. Clinically, it is therefore very important to understand its risk factors and instigate the appropriate management actions. However, of all the pregnancy complications potentially associated with PCOS, its association with fetal growth abnormalities during pregnancy appears to be the least studied, with inconclusive results. For example, in a cohort study [64] of 459 women with PCOS and a background population of 5409 women, published in 2014, obstetric outcomes were extracted from national Danish registries, and the risk of small-for-gestational-age offspring was similar in all groups. Similarly, a systematic review and meta-analysis published in 2016 [77] that included 40 observational studies reporting on 17,816 PCOS pregnancies and 123,756 non-PCOS pregnancies, found that PCOS in pregnancy did not have an impact on the risk of fetal growth abnormalities. More recently, Zhexin et al. in 2022 [78] investigated 1376 patients with PCOS and 1376 control women who had undergone invitro-fertilization/intracytoplasmic sperm injection and conceived with singleton pregnancies. PCOS patients were found to have a statistically significant lower birthweight compared with controls. PCOS women, however, also had a statistically significant higher body mass index, again raising the question about the potential confounding impact on the pregnancy outcomes in PCOS.

On the other hand, Han et al. in 2011 [29] investigated 336 women with PCOS undergoing assisted reproduction (controlled ovarian hyperstimulation with IVF or superovulation) with 1,003 infertile women who had the tubal factor as an indication for assisted reproduction treatment acting as controls and found that the incidence of small-for-gestational-age infants was higher in the PCOS groups than the tubal factor groups. Han et al. specifically found that the rate of SGA in neonates was higher in incidence in both obese PCOS and non-obese PCOS patients when compared to their non-PCOS counterparts, implying that this complication in pregnancy may be unrelated to maternal weight and more specific to PCOS or its other sequelae. Similarly, a meta-analysis published in the same year (2011) [62] reported an almost 2.6-fold increased risk of small-for-gestational-age (SGA) at birth. PCOS has also been associated with large-for-gestational-age babies and fetal macrosomia in a retrospective study [28] of the pregnancy, delivery, and neonatal outcome in overweight versus normal weight women with PCOS by De Frene et al. noting that pregnant, overweight women with PCOS had an elevated risk for LGA when compared to normal weight mothers with PCOS. 

In the first study of its kind, in PCOS, in 2021 [79] Zhang et al. retrospectively looked at the images from fetal MRIs from 60 pregnant women with PCOS (PCOS group) and 120 healthy pregnant women without PCOS (control group). The results showed that fetuses born to women with PCOS had smaller biparietal diameters, femur length, head circumference, and smaller placental thickness compared to those in the control group. There were, however, no significant differences in birth weight, maximum and minimum Biparietal diameters, or the incidence of placental abnormalities between the groups. Mothers with PCOS also had a significantly higher pre-pregnancy BMI than controls, which again raises the question about the potential confounding impact of BMI on pregnancy risk in PCOS.

## 7. Obesity and Pregnancy Complications

As obesity frequently appeared as a main confounder in most of the described conditions, we went further to investigate the relationship between obesity and the described pregnancy complications. We therefore performed a literature search in November 2022 of PubMed using the following keywords “pregnancy complications” AND “obesity”. We limited the search to systematic reviews of the literature in the last year. We identified 56 papers in the initial search, of which 2 papers were thought to be relevant.

The first study [80], was a systematic review of 7894 prospective or retrospective cohort studies exploring predictors of adverse outcomes among pregnant women living with obesity. The results showed that pre-pregnancy type 1 diabetes, non-White ethnicity, specific groups of maternal age (<20 years and ≥35 years), abdominal adiposity obesity, and history of bariatric surgery were found to increase the risks of preeclampsia, low birth weight/small-for-gestational-age, preterm birth, gestational diabetes mellitus, and stillbirth. Abdominal adiposity obesity is a key feature of PCOS. Interestingly, one of the studies [81] included in the systematic review specifically looked at the focal risk of obesity associated with PCOS and obesity type (android versus gynoid) and found an association with preeclampsia, gestational diabetes and preterm birth but not with stillbirth or low birth weight. The second study [82] was a systematic review which aimed to identify early pregnancy measures of adiposity associated with adverse maternal health outcomes. Seventy studies were included with a pooled sample of 89,588 women. A meta-analysis showed significantly increased odds of gestational diabetes mellitus (GDM) with higher waist circumference (WC) categories and per unit increase in WC. WC was also significantly associated with hypertensive disorders and delivery-related outcomes. Waist to hip ratio was also significantly associated with GDM, hypertensive disorders, and delivery-related outcomes. Increased waist circumference and WHR were both associated with PCOS.

Although the two studies above identified demographic and phenotypic features in obese patients associated with pregnancy risks, these features on their own do not explain the mechanisms. However, the paper by Fakhraei et al. [80] suggested that there was increasing evidence to implicate obesity-associated low-level inflammatory mediators as important contributors to these risks, linking maternal obesity and/or an obesogenic diet with altered adipokine secretion, insulin resistance, and increased circulating lipids, which in turn are associated with increased levels of markers of inflammation (including interleukin-, interleukin-1β, and tumor necrosis factor-α). Several of these markers of inflammation which are also elevated in PCOS have been associated with placental inflammation, altered placental nutrient transport, and altered placental structure, all of which have the potential to increase the risks of preeclampsia, fetal growth restriction, preterm birth, and stillbirth in PCOS.

These inflammatory mediators might therefore provide a mechanistic basis for some of the observed associations between PCOS and pregnancy complications identified in our narrative review. However, more studies are required given the complexity of the PCOS phenotypes.

## 8. Conclusions

The challenge of PCOS clearly does not end at conception. Our narrative review has highlighted the fact that the incidence of adverse outcomes for women with PCOS during pregnancy including spontaneous miscarriage, gestational diabetes mellitus, hypertension in pregnancy, and preterm birth may be increased. However, the rates appear to differ according to the features of PCOS present, and most importantly, the impact of the confounding effect of maternal obesity in PCOS on these adverse outcomes needs clarification. This makes PCOS a challenging disease that not only affects a woman’s ability to conceive but also appears to have consequences throughout her pregnancy.

From a practical perspective, regardless of the currently unanswered research questions, the most pragmatic approach might be to develop specific protocols for PCOS mothers for follow-up during their pregnancy (Table 2).

For example, within the umbrella of pregnant PCOS patients, there are those that suffer from obesity and would likely be at an elevated risk for GDM, and there are also those with hyperandrogenemia, which might put them at a higher risk for preeclampsia. It might be reasonable to consider early screening for GDM and hypertension in all pregnant PCOS women with even closer follow-up for those with a pre-pregnancy BMI > 25. The relationship between maternal PCOS and fetal growth abnormalities may be somewhat debatable, but increased caution on the matter and added screening might be justifiable. There is also the likelihood that PCOS patients may suffer from cervical insufficiency and in turn have higher rates of preterm deliveries, and further investigations during pregnancy might therefore be justified to prevent this risk.

From a research perspective, larger global and multi-center epidemiological studies, carefully controlling for potential confounding variables such as obesity and accounting for the various PCOS phenotypes, would be useful to clarify the exact risks of pregnancy complications in PCOS. New or ongoing birth cohort studies might provide an excellent resource to address this important gap in the literature and investigate the potential role of novel molecular markers in mediating the pregnancy risks in PCOS such as co-enzyme A restriction in mediating the increased preeclampsia risk in PCOS.

## Figures and Tables

**Table 1:** Potential pregnancy complications in PCOS and possible mechanisms.

Complication	Possible Mechanisms
Spontaneous miscarriage	Obesity [13], LH hypersecretion [14], hyperhomocysteinaemia [15], decreased plasminogen activator inhibitor-1 activity (PAI-1) and endothelial dysfunction [16], and vitamin D deficiency and vitamin D-binding protein [17].
Gestational diabetes	Obesity [18], maternal insulin resistance [19], and low vitamin D levels [20].
Hypertension in pregnancy	Obesity [21], insulin resistance [22], hyperhomocysteinaemia [23] and co-enzyme A restriction [24].
Preterm birth	Obesity [25], increased incidence of multiple pregnancies [18], nulliparity, increased estrone levels, hyperinsulinemia, requirement for assisted reproductive technology treatment [26], hypertensive disease [26], cervical insufficiency increased levels [27].
Fetal growth abnormalities	Uncertain. Maternal obesity has been associated with both fetal macrosomia [28] and small-for-gestational-age babies [29] in PCOS.

**Table 2:** Potential pregnancy complications in PCOS and implications for clinical practice.

Complication	Pragmatic Considerations for Clinical Practice
Spontaneous miscarriage	Advise weight reduction prior to pregnancy.
Gestational diabetes	Advise weight reduction prior to pregnancy. Early screening for gestational diabetes.
Hypertension in pregnancy	Advise weight reduction prior to pregnancy. Early screening for hypertension in pregnancy, consider low-dose aspirin.
Preterm birth	Advise weight reduction prior to pregnancy, consider ultrasound scans for cervical length measurements.
Fetal growth abnormalities	Advise weight reduction prior to pregnancy, regular fetal growth scans, and consider ultrasound scans for cervical length measurement.

## Data Availability

Not applicable.
